# Discovery of Two Novel Oxidases Using a High‐Throughput Activity Screen

**DOI:** 10.1002/cbic.202100510

**Published:** 2021-11-18

**Authors:** Elzbieta Rembeza, Alessandro Boverio, Marco W. Fraaije, Martin K. M. Engqvist

**Affiliations:** ^1^ Department of Biology and Biological Engineering Chalmers University of Technology 412 96 Gothenburg Sweden; ^2^ Molecular Enzymology Group University of Groningen Nijenborgh 4 9747AG Groningen The Netherlands

**Keywords:** enzyme discovery, flavoproteins, high-throughput screening, orphan enzymes, oxidoreductases

## Abstract

Discovery of novel enzymes is a challenging task, yet a crucial one, due to their increasing relevance as chemical catalysts and biotechnological tools. In our work we present a high‐throughput screening approach to discovering novel activities. A screen of 96 putative oxidases with 23 substrates led to the discovery of two new enzymes. The first enzyme, N‐acetyl‐D‐hexosamine oxidase (EC 1.1.3.29) from *Ralstonia solanacearum*, is a vanillyl alcohol oxidase‐like flavoprotein displaying the highest activity with N‐acetylglucosamine and N‐acetylgalactosamine. Before our discovery of the enzyme, its activity was an orphan one ‐ experimentally characterized but lacking the link to amino acid sequence. The second enzyme, from an uncultured marine euryarchaeota, is a long‐chain alcohol oxidase (LCAO, EC 1.1.3.20) active with a range of fatty alcohols, with 1‐dodecanol being the preferred substrate. The enzyme displays no sequence similarity to previously characterised LCAOs, and thus is a completely novel representative of a protein with such activity.

## Graphical abstract

**In search of novel enzymatic activities**: Application of a high‐throughput activity screening platform has led to the discovery of two novel enzymes: N‐acetyl‐hexosamine oxidase, a previously orphan enzyme, and a novel type of long‐chain alcohol oxidase, a non‐homologous isofunctional enzyme (NISE) to known enzymes with this activity.

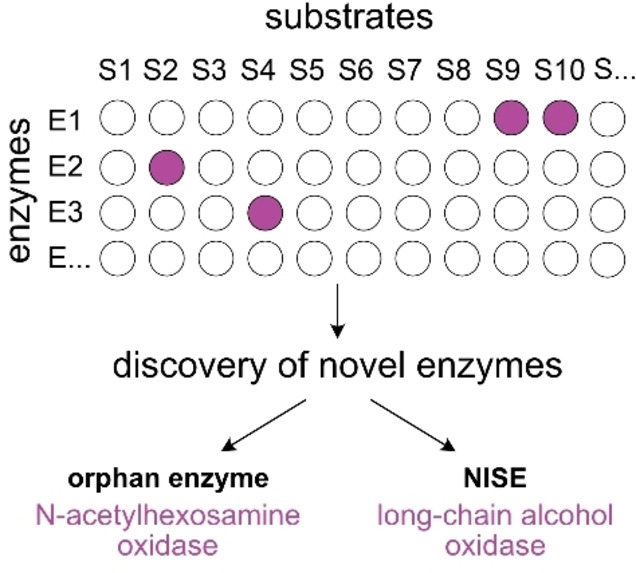

## Introduction

Enzymes are macromolecules that catalyse chemical reactions in living organisms. Years of enzyme studies have provided great insight into their significance in metabolism and disease.^[1]^ Many enzymes are also indispensable in food, agriculture, chemical and pharmaceutical industries.[^2^, ^3^] Finding novel enzymatic activities, however, remains a challenging task.

Although the number of potential enzyme sequences in sequence databases is growing rapidly, not every gene can confidently be annotated based on its corresponding protein sequence alone. As a result, various experimental approaches have been undertaken to find new enzyme activities.[^4^, ^5^, ^6^, ^7^, ^8^, ^9^, ^10^] Family‐wide substrate profiling has led to discovery of various enzyme activities and allowed for a deeper understanding of sequence‐function relationships within the families.[^4^, ^5^, ^6^] This approach, however, by design focuses on exploration of activities in homologous enzymes. Functional screens of diverse sequence libraries, applied oftentimes in metagenomic screening, proved useful in finding new enzymes that lack homology to known sequences.[^7^, ^8^] A downside of such screens is their limited focus, as they aim to detect only specific enzyme activities of interest. Nontargeted *in vitro* metabolomics is an example of an approach that enables screening for a wide range of activities. This method resulted in annotation of many enzymes with unknown functions, although labelling the majority of metabolites without ambiguity proves difficult.[^9^, ^10^] Each approach to finding new enzymatic activities has its advantages and disadvantages; together with the ever‐evolving bioinformatic tools, they complement each other in this challenging task.[^11^, ^12^, ^13^]

Oxidases acting on the CH‐OH group of electron donors with dioxygen as acceptor (EC 1.1.3.X) are a diverse group of enzymes present predominantly in bacteria, fungi, and plants. They take part in a wide range of biological processes, such as photorespiration^,^ production of osmoprotectants, synthesis of antibiotics and phytotoxins.[^14^, ^15^, ^16^, ^17^, ^18^] Most of the enzymes annotated to these oxidases are flavoenzymes, consuming dioxygen and producing hydrogen peroxide. Representatives of the 1.1.3.X oxidases are of particular interest to the medical and food industries as biosensor candidates.[^19^, ^20^] In such biosensors, direct detection of electrons or monitoring formation of hydrogen peroxide allow the measurement of specific marker molecules, such as glucose, lactate, ethanol, cholesterol, galactose, and choline. Glucose oxidase‐based glucometers are currently the most commonly used point‐of‐care biosensors; the development of new biosensors that could match its potential requires extensive research into new, stable, easy to produce enzymes. Oxidases annotated to 1.1.3.X are also important tools in organic synthesis, being used in production of drugs, antioxidants, flavour, and fragrance compounds.[^21^, ^22^] Nearly 15000 sequences are currently annotated as 1.1.3.X in UniProt (https://www.uniprot.org), but only a fraction of them have been experimentally characterised. With their potential applications in mind, investigation of a wider swath of these oxidases would be highly beneficial.

Computational methods of function annotation have their limits, particularly when the sequence similarity between experimentally characterized proteins and newly sequenced ones grows. Annotation inaccuracies are common, and for many sequences it is difficult to computationally assign specific substrates, or predict a side activity with certainty.[^23^, ^24^] In this work we worked under the hypothesis that, due to annotation errors, novel enzyme activities might be found among sequences already annotated to an enzyme class. We explored the activity profile of sequences annotated as 1.1.3.X oxidoreductases by using a large‐scope, high‐throughput screening approach.

Among 96 tested sequences, we found two novel enzymes: an orphan enzyme, N‐acetyl‐D‐hexosamine flavoprotein oxidase, and a long‐chain alcohol flavoprotein oxidase of a novel kind. We characterised the novel oxidases, defined their substrate scope, performed sequence analysis, and investigated possible biological functions. Overall, we present a successful approach for the discovery of novel enzymes.

## Results and Discussion

### High‐throughput screen of EC 1.1.3.X enzymes

In order to gain insight into the activity profile of oxidoreductases, we performed an “all‐vs‐all” experiment, where representative enzymes annotated as EC 1.1.3.X were assayed with 23 representative substrates selected from each of the EC 1.1.3.X enzyme classes. In our setup 96 sequences were chosen, containing proteins annotated to 12 different oxidoreductase enzyme classes (Table S1). The genes were chosen to uniformly represent the sequence space of EC 1.1.3.X, and their number adjusted to fit into a 96‐well microwell expression plate (Computational Methods, Sequence selection). Selected genes were synthesized, expressed in *Escherichia coli*, and the obtained proteins were purified and assayed for oxidase activity with the 23 representative substrates (Table S2). A total of 56 enzymes were successfully purified, but 46 of them displayed no activity, even with their predicted substrate (Table S1). This finding led us to investigate the issue of protein mis‐annotation in enzyme databases, which we covered in our previous work.^[24]^ Only ten of the purified enzymes were active with one or more substrates; eight were active with substrates of the ECs to which they were annotated, while two enzymes were active with a substrate of another EC class (Table S1).

The first of these, A3RXB7 from *Ralstonia solanacearum UW551*, was annotated as aclacinomycin‐N oxidase (EC 1.1.3.45) but displayed activity with N‐acetyl‐D‐glucosamine (EC 1.1.3.29). The second protein, A0A075HNX4 from an uncultured marine euryarchaeota, was annotated as an L‐gulonolactone oxidase (EC 1.1.3.8) but displayed activity with dodecanol (EC 1.1.3.20). The former protein is of particular interest, as EC 1.1.3.29 is an orphan activity ‐ an enzyme activity that has been experimentally characterized but for which there is no sequence information.^[25]^ Enzymes with the long chain alcohol oxidase (EC 1.1.3.20) activity have been described before, but never from an archaeal host and only from sequence‐unrelated flavoproteins.^[26]^ We decided to perform a detailed characterisation of the two newly identified oxidases.

### Characterisation of N‐acetyl‐D‐hexosamine oxidase from R. solanacearum

#### biochemical characterisation

The first enzyme displaying N‐acetyl‐D‐hexosamine (HexNAc) oxidase activity was originally purified from a *Pseudomonas*‐like bacterium, however, no sequence information was linked to the activity.^[25]^ The enzyme was reported to be active on a range of monosaccharides, and displayed the highest affinity to N‐acetyl‐D‐glucosamine (GlcNAc) and N‐acetyl‐D‐galactosamine (GalNAc). We purified the *R. solanacearum* A3RXB7 enzyme, performed kinetic experiments on a range of sugar substrates, and compared the resulting activities with the published values of the reported *Pseudomonas* enzyme. Our results show a very similar activity profile of the two enzymes (Table 1, Table S3). Both enzymes are active on a range of monosaccharides, with highest affinity towards GlcNAc and GalNAc. Both proteins are also active with chitobiose, the disaccharide of GlcNAc, although with less affinity than the monomer itself. These results strongly support our discovery of a sequence for the orphan activity of N‐acetyl‐D‐hexosamine oxidase (EC 1.1.3.29). The biggest difference between the two proteins is their affinity towards D‐glucosamine and D‐galactosamine: nearly an order of magnitude higher for the *R. solanacearum* A3RXB7 (Table 1). These differences indicate that although the two proteins display a similar activity profile, their function and mode of action might be different.

**Table 1:** Comparison of substrate specificity of the N‐acetyl‐D‐hexosamine oxidases characterised in the paper first describing the activity (Horiuchi, 1989) and in our paper (A3RXB7).

Substrate	K_M_ [mM]		Specific activity [μmol min^−1^ mg^−1^]	
	Horiuchi, 1989^[25]^	A3RXB7	Horiuchi, 1989^[25]^	A3RXB7
GlcNAc	0.24	0.26	71	6.08
GalNAc	0.1	0.32	70	4.78
ManNAc	40	182	12	2.39
chitobiose	18	19	45	0.9
D‐glucosamine	40	4.5	34	0.38
D‐galactosamine	10	1.4	35	0.67
D‐mannosamine	–	65	–	0.01
D‐glucose	290	216	3.8	0.17
D‐galactose	170	102	3.3	0.21
D‐mannose	59	118	1.2	0.15

**Figure 1:**
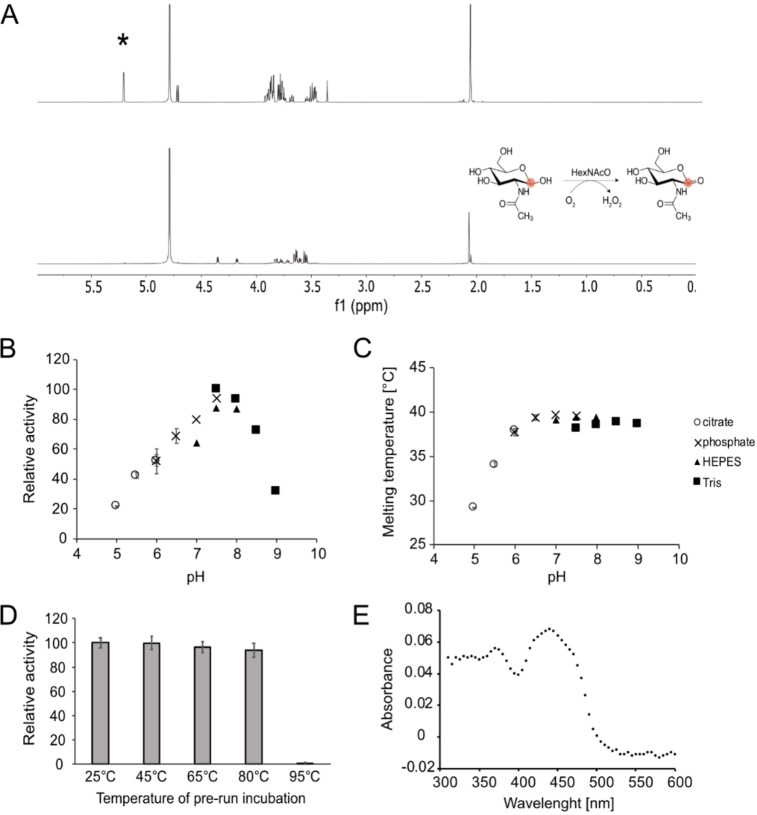
Characterisation of HexNAcO from *R. solanacearum*. (A) ^1^H‐NMR spectra of GlcNAc (upper panel) and GlcNAc after incubation with the HexNAcO oxidase (lower panel). The star indicates a peak corresponding to the hydrogen of a hydroxyl group by the C1 carbon of GlcNAc. The diagram illustrates the reaction catalysed by N‐acetyl‐D‐hexosamine oxidase on the example of GlcNAc as a substrate, with carbon C1 circled in red. (B) Screen of HexNAcO activity in a range of buffers. (C) Screen of HexNAcO melting temperature in a range of buffers. (D) Activity of HexNAcO with GlcNAc upon 30 minute pre‐incubation in a range of temperatures. (E) UV/Vis absorbance spectrum of purified HexNAcO. When applied, the error bars display standard deviations for three replicates.

Further investigation with proton nuclear magnetic resonance revealed that the HexNAc oxidase from *R. solanacearum* acts upon the C1 carbon of the GlcNAc molecule, producing the corresponding lactone (Figure 1A). The pH optimum of the enzyme was 7.5 (Figure 1B), and its melting temperature was 39 °C in buffers with pH 6.5 and above (Figure 1C), although the enzyme was able to refold and regain its activity upon heating to up to 80 °C (Figure 1D). The purified protein is yellow, and its absorption spectrum displayed maxima at 375 and 445 nm (Figure 1E), which is characteristic for a flavoprotein. Although the protein was produced in high amounts (25 mg/l), its expression caused growth inhibition of the *E. coli* host cells upon induction (Figure S1). The growth inhibition was not caused by the induction itself, as expression of a control oxidase enzyme, lactate oxidase from *Streptococcus shiloi*, did not cause any growth inhibition (Figure S1A). It is likely that the overexpressed enzyme reacts with intracellular GlcNAc destined for the host cell walls, which causes hydrogen peroxide accumulation, and as a result inhibition of cell growth and inefficient heterologous gene expression.

### Comparison of HexNAc oxidase to other vanillyl alcohol oxidase‐like enzymes

**Figure 2:**
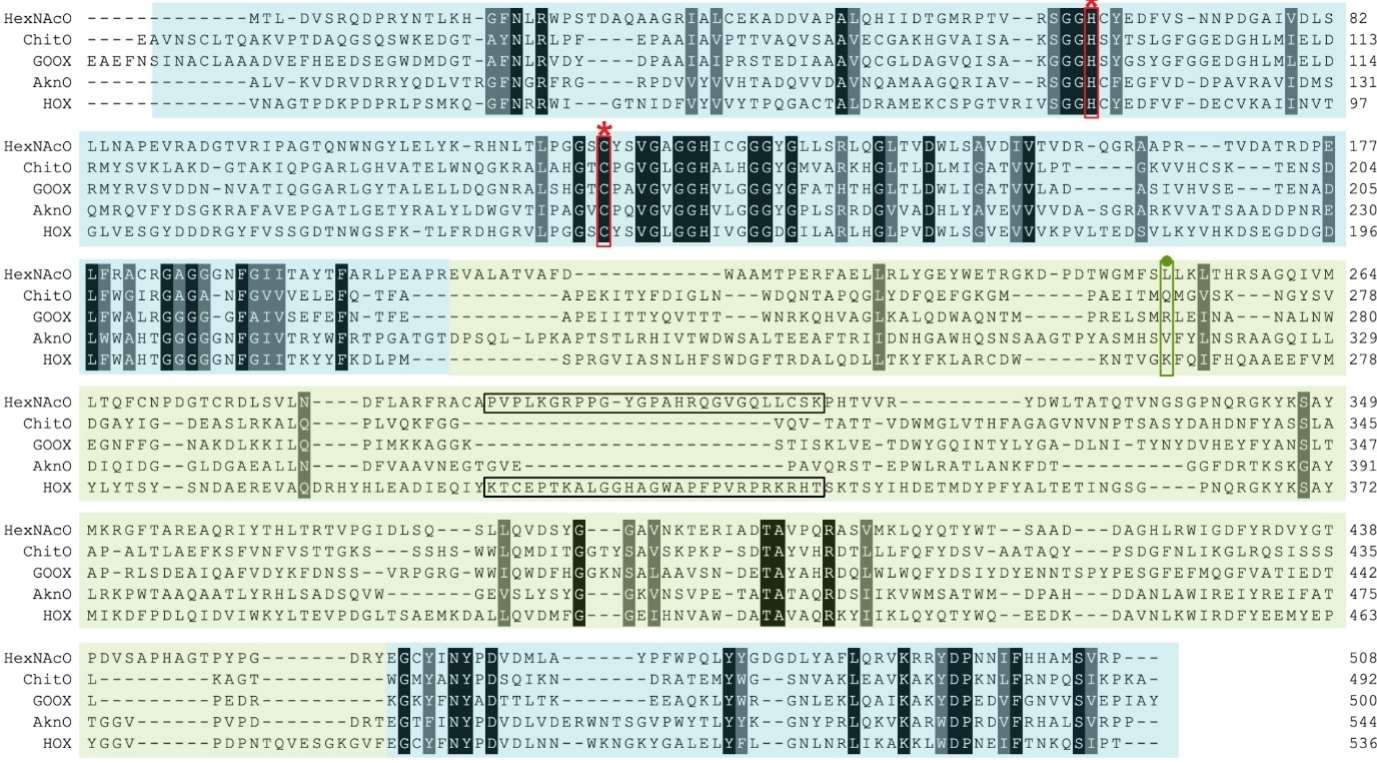
Structure‐based multiple sequence alignment of representatives of characterised VAO type‐enzymes and HexNAcO characterised in this study. ChitO: chitooligosaccharide oxidase from *Fusarium graminearum*, GOOX: glucooligosaccharide oxidase from *Acremonium strictum*, AknO: aclacinomycin oxidase from *Streptomyces galilaeus*, HOX: hexose oxidase from *Chondrus crispus*. Highlighted in blue is the FAD‐binding domain and in green the substrate binding domain. Histidine and cysteine residues conserved are marked with red stars. Residues corresponding to Gln268 in ChitO in homology models are marked with a green dot. Residues in HexNAcO and HOX forming a loop at the substrate cavity are circled in black. Identical residues in the homologues are highlighted in black, similar residues are highlighted in grey. MSA created with PROMALS3D.^[28]^

In order to learn more about the sequence‐function relationship of the newly found HexNAcO, we searched for its closest related experimentally characterised proteins. The search revealed the highest identity to bacterial oxidases: tirandamycin oxidase (TamL, D3Y1I2) from *Streptomyces sp*. (37.4 %), hexose oxidase (Dbv29, Q7WZ62) from *Nonomuraea gerenzanensis* (37.4 %), aclacinomycin oxidase (AknO, Q0PCD7) from *Streptomyces galilaeus* (35.1 %), as well as eukaryotic hexose oxidase (HOX, P93762) from alga *Chondrus crispus* (35.3 %). The four homologous enzymes are all berberine bridge enzyme (BBE)‐like enzymes, which display a vanillyl alcohol oxidase (VAO) fold and contain a bicovalently anchored FAD cofactor.^[27]^ Members of this family of enzymes contain a well‐conserved FAD binding domain spanning N‐ and C‐termini, and a central, more variable substrate binding domain. Multiple sequence alignment of HexNAc oxidase with other VAO‐type enzymes reveals a similar architecture (Figure 2). The FAD‐anchoring histidine and cysteine are conserved, responsible for the bicovalent binding of FAD: His64 and Cys123 (Figure 2).

Among the VAO‐type oxidases there is a number of characterised carbohydrate oxidases, most of them eukaryotic, acting on both mono and disaccharides (hexose oxidase), as well as on oligo and polysaccharides (glucooligosaccharide oxidase (GOOX), xylooligosaccharide oxidase (XylO), or chitooligosaccharide oxidase (ChitO)).^[29]^ ChitO is an enzyme with the closest substrate profile to HexNAcO ‐ acting on N‐acetylated sugars. However, unlike HexNAcO, it displays more affinity towards its polysaccharides.^[30]^ Gln268 was recognised as a crucial residue for operating on N‐acetylated substrates in ChitO, being smaller than an arginine residue present in other saccharide oxidases acting on nonacetylated substrates.^[30]^ Based on a structure prediction model of HexNAcO, no glutamine residue is present in the vicinity, but rather a leucine (Leu251) which, like glutamine, is also smaller than an arginine residue (Figure 2).

**Figure 3:**
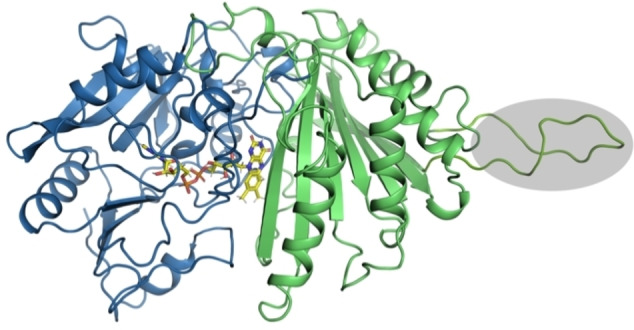
Homology model of HexNAcO. FAD‐binding domain is coloured in blue, substrate binding domain is coloured in green, highlighted in grey is the elongated loop of the substrate binding domain. Model obtained using RaptorX server, using 2Y08 chain A structure (tirandamycin oxidase TamL) as a template.

Multiple sequence alignment and a homology model of HexNAcO reveal a unique feature in its substrate binding domain ‐ an elongated stretch of amino acids (amino acids 293–318, approximately), possibly forming a loop by the edge of the substrate binding cavity (Figure 2, Figure 3). A similar extension of the substrate binding domain can also be found in another carbohydrate oxidase operating primarily on monosaccharides: hexose oxidase from *C. crispus*. It is possible that such a loop acts as a lid of the substrate binding domain, allowing access of primarily smaller substrates. Elucidation of the structure of HexNacO is needed to confirm the existence of the loop, and would allow for a more detailed analysis of the function of the loop, as well as the whole enzyme.

### Exploration of HexNAcO function

The biological function of HexNAcO from *R. solanacearum* is unclear, as the organism has no known pathways or mechanisms involving oxidation of N‐acetyl‐hexosamines. Like other characterised carbohydrate oxidases, its main function might be connected to extracellular production of hydrogen peroxide to compete with other organisms through oxidative stress, or support the action of peroxidases and peroxygenases.^[29]^ This would be possible via oxidising N‐acetyl hexosamines present in the environment as components of chitin and bacterial exopolysaccharides. N‐acetylglucosamine, as a potential substrate, is also present inside bacterial cells as a product of cell wall's peptidoglycan recycling. However, intracellular peroxide producing oxidases in microorganisms are rare, as they lead to accumulation of reactive oxygen species. Previous studies revealed that HexNAcO is a non‐essential gene, as indicated by the transposon essentiality experiments in four *R. solanacearum* strains, in rich and minimal media.^[31]^ Additionally, expression of the gene was shown to be among the ones positively regulated by exopolysaccharide production and quorum sensing, which might suggest that HexNAcO is involved in a biofilm formation process.^[32]^

The closest homologues of HexNAcO from *R. solanacearum* (down to 50 % sequence identity) come from Gram negative (G‐) bacteria of *Acidobacteriales* and *Burkholderiales* order (Figure S2), most commonly inhabiting soil and water habitats. These organisms are poorly characterised, with *R. solanacearum*, a plant pathogen, being the best characterised organism that contains an HexNAcO homologue. Some of the species containing the gene are characterised by growth in low nutrient and highly contaminated environments (*Ralstonia pickettii*, *Variovorax paradoxus*), and displaying plant growth promoting capabilities (*V. paradoxus*).[^33^, ^34^] Further molecular and physiological studies of soil microorganisms would be required to help elucidate what function HexNAcO, and its homologs, play in those organisms.

### Characterisation of novel archaeal long chain alcohol oxidase

Protein A0A075HNX4 from uncultured marine group II/III euryarchaeota KM3_72_H01 (*Eur*) was annotated in BRENDA DB (https://www.brenda‐enzymes.org) as L‐gulonolactone oxidase (EC 1.1.3.8). However, in our “all‐vs‐all” experiment, the protein showed activity with 1‐dodecanol, which is characteristic for long chain alcohol oxidases (EC 1.1.3.20). Long chain alcohol oxidases (LCAOs) catalyse oxidation of alcohol substrates with carbon chain lengths of six and above, usually accepting a wide range of fatty alcohols. Characterised LCAOs have been reported from plant species (*Simmondsia chinensis, Arabidopsis thaliana, Lotus japonicus*) and fungi (*Candida tropicalis, Candida bombicola, Yarrowia lipolytica, Aspergillus terreus*).^[26]^ LCAOs have been shown to participate in fatty acid metabolism via an ω‐oxidation pathway, enabling growth on fatty acids/alkanes in yeast and mobilisation of seed storage reserves in plants.[^35^, ^36^] LCAOs are predominantly membrane‐bound, localising to mitochondria, microsomes, and glyoxysomes.^[26]^

### Biochemical characterisation

**Figure 4:**
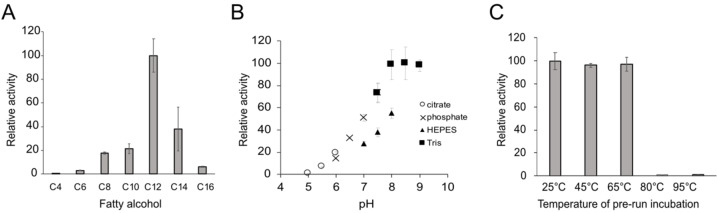
Characterisation of LCAO from uncultured marine group II/III euryarchaeote. (A) Activity of LCAO with a range of fatty alcohols (C4: 1‐butanol, C6: 1‐hexanol, C8: 1‐octanol, C10: 1‐decanol, C12: 1‐dodecanol, C14: 1‐tetradecanol, C16: 1‐hexadecanol). (B) Activity of LCAO with 1‐dodecanol in a range of buffers. (C) Activity of the LCAO with 1‐dodecanol upon 30 min. incubation in a range of temperatures. Error bars display standard deviation for three replicates.

In order to confirm the catalytic function of A0 A075HNX4 as LCAO, we purified the protein and tested its activity with a range of fatty alcohols. The protein was active with substrates having a carbon chain length of six and above, showing the highest activity with 1‐dodecanol (Figure 4A). Kinetic experiments for 1‐dodecanol were carried out, resulting in a *K*_M_ value of 27.7 μM (+/−3.8 μM) and specific activity of 0.23 μmol min^−1^ mg^−1^. Both of these values are comparable to the ones listed in the BRENDA enzyme database for previously characterised LCAOs, where the *K*_M_ for 1‐dodecanol as the main substrate ranges 4–60 μM and specific activity ranges 0.02–0.22 μmol min^−1^ mg^−1^. These results confirm that A0A075HNX4 is indeed acting as a bona fide long chain alcohol oxidase, and the first one reported from an archaeal species. Further characterisation of the enzyme shows it has the highest activity above pH 8, and almost no activity at pH 5 (Figure 4B). A melting temperature of the protein was not established, as the protein was not compatible with the thermofluor assay, but the enzyme was able to refold and regain its activity upon heating up to 65 °C (Figure 4C).

### Sequence analysis

**Figure 5:**
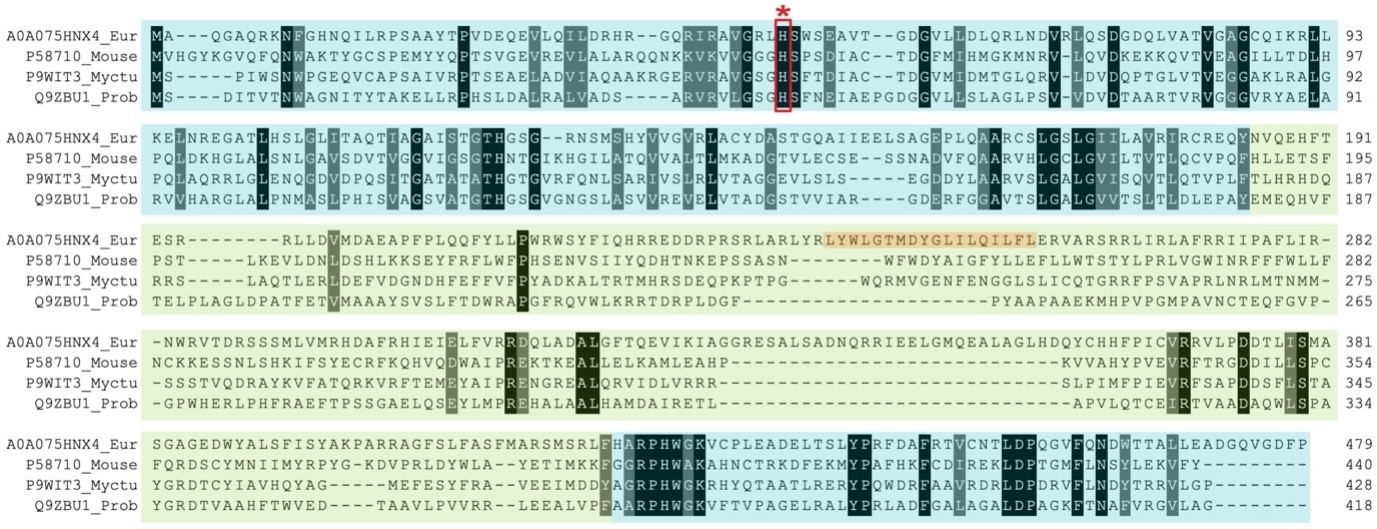
Multiple sequence alignment of archaeal LCAO and its characterised homologues. A0A075HNX4_Eur: long chain alcohol oxidase from uncultured marine group II/III euryarchaeota, P58710_Mouse: L‐gulonolactone oxidase from mouse, P9WIT3_Myctu: L‐gulonolactone dehydrogenase from *Mycobacterium tuberculosis*, Q9ZBU1: xylitol oxidase from *Streptomyces coelicolor*. Highlighted in blue is the FAD‐binding domain, in green substrate binding domain, in orange the predicted membrane binding motif of archaeal LCAO. Marked with a red star is a conserved histidine residue responsible for covalent binding of the FAD cofactor. Identical residues in the homologues are highlighted in black, similar residues are highlighted in grey. MSA created with PROMALS3D.

**Figure 6:**
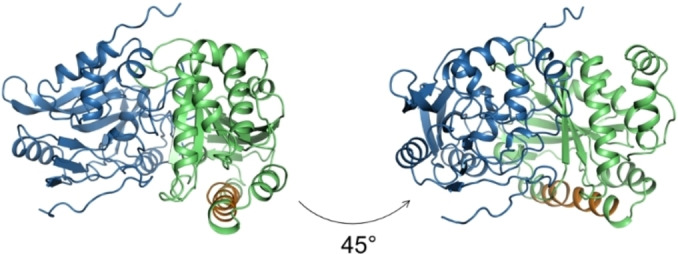
Structural model of LCAO. The PCMH‐type FAD‐binding domain is coloured in blue, the substrate binding domain is coloured in green, the membrane‐bound helix is coloured in orange. Model obtained using AlphaFold Collab.^[39]^

Although the newly discovered archaeal LCAO displays the same activity profile as previously characterised LCAOs, it shares no sequence similarity with them. Known LCAOs belong to the glucose‐methanol‐choline (GMC) superfamily of oxidoreductases (IPR012132), composed of an N‐terminal FAD‐binding domain containing a Rossmann fold, and a C‐terminal substrate binding domain.^[37]^ In contrast, the InterPro scan of the LCAO sequence from *Eur* revealed that the enzyme resembles a FAD‐containing lactone oxidase (ALO family, IPR010031), being a member of the VAO flavoprotein family.^[27]^ It is predicted to contain an FAD‐binding domain, which spans the N‐ and C‐termini of the protein and consists of two alpha‐beta subdomains accommodating the FAD cofactor (Figure 5, Figure 6). The histidine residue responsible for covalent binding of the flavin cofactor in the FAD‐binding domain is conserved in the LCAO (His 49, Figure 5).

A majority of known LCAOs are membrane‐bound, and the *Eur* LCAO sequence also contains a predicted membrane binding helix within the substrate binding domain (Figure 5, Figure 6). Based on the positioning of the helix, the protein is likely a monotopic membrane protein, attaching to the membrane from one side (Figure 6). The closest characterised enzymes to the *Eur* LCAO, in terms of sequence identity, are L‐gulonolactone dehydrogenase from *Mycobacterium tuberculosis* (27 % sequence identity) and mouse L‐gulonolactone oxidase (25 % seq. identity), which might explain why the protein was annotated as L‐gulonolactone oxidase (EC 1.1.3.8). Overall, the sequence analysis of the archaeal LCAO revealed that the enzyme is a truly novel fatty alcohol oxidase. The previously described fungal and plant LCAOs (GMC superfamily members) and the novel archeal LCAO (VAO family member) are thus an example of non‐homologous isofunctional enzymes: evolutionarily unrelated enzymes that catalyse the same chemical reaction.^[38]^

The closest uncharacterized homologues of *Eur* LCAO come from archaeal and bacterial hosts inhabiting marine environments (Figure S3). Both naturally produced and man‐made lipids are present in the aquatic environment and can act as a source of energy.[^40^, ^41^] It is therefore possible that LCAO of marine microorganisms takes part in their metabolism, in a pathway similar to the ω‐oxidation pathway described for yeast.^[35]^

## Conclusions

In this work we discovered two novel enzymes: one orphan enzyme, for which only the activity, but not the sequence was known, and one non‐homologous isofunctional enzyme, performing a known activity, but being evolutionarily unrelated to previously described representatives. Both of these enzyme types were already annotated to other enzyme classes, but those activities were not present in the two novel enzymes. It is worth noting, that although the enzymes display an *in vitro* oxidase activity, their *in vivo* function might be that of a dehydrogenase, using an electron acceptor alternative to oxygen, for instance a quinone. The ability of both of these enzymes to act as oxidases, makes them good potential candidates for various applications. HexNAcO from *R. solanacearum* might be used in a screen detecting chitin degrading enzymes, similar to the one described for chitooligosaccharide oxidase.^[42]^ Upon modification of substrate specificity, through protein engineering, HexNAcO might also display other promising carbohydrate‐processing activities. Oxidation of fatty alcohols by LCAOs is applied in the production of dicarboxylic acids used as precursors of polyamides, polyesters, perfumes, plasticizers, lubricants, and adhesives.^[43]^ Since the novel archaeal LCAO is predicted to have a different fold from the previously known representatives, it could be an interesting alternative candidate for biosynthesis of dicarboxylic acids. The novel LCAO might also be used as a fatty alcohol biosensor, or applied in screening for new fatty alcohol synthesizing enzymes.

In order to find these novel activities, we applied a high‐throughput screening approach by testing nearly a hundred enzymes with 23 different substrates. This screening approach provides a good setup for finding activities of enzymes with low or no homology to known sequences. By testing a range of very different substrates, we widened the possibility of detecting activities that would otherwise be impossible to annotate by family‐wide screening or homology‐based bioinformatic approaches. Application of our approach to other enzyme classes would likely uncover additional novel enzymes. We also believe that this setup could be well‐suited for discovery of promiscuous activities ‐ side reactions that are distinct from the enzyme‘s main activity ‐ which are currently of increasing interest in biological research.^[44]^

There is considerable room for further building on the approach used in this study. One of the main limits of the current approach is its focus on only one type of reaction that is being screened: oxidase activity assayed by detection of hydrogen peroxide. It is possible that some enzymes in the selection display an altogether different chemistry, but the setup we applied would not allow us to detect them. Additionally, the type of substrates tested was limited to the ones commercially available, and their number limited to suit the format of a 384‐well plate. Finally, the screen focused on testing successfully expressed and purified enzymes (recovery of 58 %) is yet another limitation. Future improvements on the approach would need to address these imitations. For instance, both the reaction and substrate limitations mentioned above might be resolved by running assays on purified proteins and using metabolites found in cell extracts as substrates, coupled with GC‐MS detection of products formed.^[10]^ Alternatively, throughput of fluorometric assays might be increased using microfluidic methods that enable automated all‐vs‐all evaluation of many enzymes and substrates.^[45]^

## Experimental Section

**Protein purification**: Representative genes of 1.1.3.X were codon optimized, synthesised, cloned into the pET21a vector and sequenced‐verified by Twist Bioscience. Between the gene sequence and vector backbone, a C‐terminal linker was added (AAALEHHHH). High‐throughput purification was carried out according to a published protocol.^[46]^ Briefly, plasmids were transformed into *E. coli* BL21(DE3) cells, and expression was carried out in 1 mL autoinduction TB medium in 96‐well deep well plates. After expression, cells were lysed, and purification in a 96‐well format was carried out, using Talon resin and 96‐well desalting plate. After purification, the proteins were analysed by SDS‐PAGE. For the preparative purification, expression was carried out in 100 mL autoinduction TB (Formedium) with 100 μg/mL carbenicillin, shaking 220 rpm in 37 °C for 4 h, followed by 18 h in 25 °C. Cells were spun down for 10 min at 5000 *g*, resuspended in 10 mL lysis buffer (50 mM HEPES pH 7.4, 5 % glycerol, 300 mM NaCl, 0.5 mM TCEP, 0.5 mg/mL lysozyme, 10 U/mL DNaseI), and incubated in room temperature for 30 min. Triton‐X‐100 was added to the final concentration of 0.125 % (HexNAcO) or 1 % (LCAO), and the lysate was frozen at −80 °C for 30 min. Lysate was thawed in a room temperature water bath and spun down for 45 min at 5000 *g*. Soluble fraction of the lysate was mixed with 1.5 mL 50 % Talon resin equilibrated in sample buffer (50 mM HEPES pH 7.4, 5 % glycerol, 300 mM NaCl, 0.5 mM TCEP), and imidazole was added to the final concentration of 10 mM. After 15 min rotating at 4 °C, the lysate‐resin mixture was applied to a glass column (BioRad, 0.5 cm diameter). Resin was washed twice with a column volume of sample buffer, and twice with a wash buffer (50 mM HEPES pH 7.4, 5 % glycerol, 300 mM NaCl, 0.5 mM TCEP, 40 mM imidazole). Protein eluted in 2.5 mL elution buffer (50 mM HEPES pH 7.4, 5 % glycerol, 300 mM NaCl, 0.5 mM TCEP, 250 mM imidazole) and desalted on a PD10 column with 3.5 mL sample buffer. After purification, the proteins were analysed by SDS‐PAGE, and their concentration measured by the Qubit protein assay. Proteins were aliquoted and stored in −80 °C for further analysis.

**Activity assays**: Amplex Red (AR)‐based oxidase activity assay was used for all the activity measurements. In the assay, AR reacts in a 1 : 1 stoichiometry with hydrogen peroxide in the presence of horseradish peroxidase (HRP) to produce highly fluorescent resorufin. The standard reaction buffer contained 50 μM AR, 0.1 U/mL HRP, buffer, protein and substrate. Standard reactions were carried in triplicates, in a black 384‐well low volume plate (Greiner), at 25 °C, with a reaction volume of 20 μL. Readouts of fluorescence were carried out with an excitation filter of 544 nm and emission filter of 590 nm in a BMG Labtech FLUOstar Omega plate reader. For the “all‐vs‐all” activity assay, 20 mM HEPES pH 7.4 buffer was used, 0.8 μL of each protein (Table S1) and 1 mM of each substrate (Table S2) were added to each reaction mixture, and endpoint measurements were recorded. Values obtained for BSA (0.5 mg/mL) were used to establish a limit of detection of the assay (mean_BSA_+5*SD_BSA_). For kinetic characterisation, measurements were taken every 20 sec for 20 minutes, HexNAcO reactions were carried in a buffer containing 20 mM HEPES pH 7.4 and 150 mM NaCl, while for LCAO reactions were carried in a buffer containing 100 mM Tris pH 8.5 and 100 mM NaCl. Resorufin standard curve measurements were used to calculate the amount of product formed, and the ICEKAT tool was used to calculate slopes of the linear range of reaction.^[47]^ For the buffer screen experiments, the buffers contained 100 mM NaCl and 100 mM of each buffer. For the measurements of thermal stability, proteins were incubated for 30 min at different temperatures (25 °C, 45 °C, 65 °C, 80 °C, 95 °C), followed by cooling down in ice for 1 min, 10 min incubation at 25 °C, and activity measurement with 0.2 mM GlcNAc for the HexNAcO and 2 mM 1‐dodecanol for LCAO. For the LCAO substrate specificity measurements, 0.1 μM of protein and 2 mM of each substrate were used.

**Melting temperature**: Thermofluor assay with a SYPRO Orange fluorescent probe was used. Reaction mixture contained 3 μM protein, x5 SYPRO Orange dye, and appropriate buffer. Standard reactions were carried in triplicates, in a white 96‐well qPCR plate, with a reaction volume of 20 μL. Reactions were carried out in the Mx3005P qPCR machine, with a temperature gradient of 1 °C per 1 min, excitation filter of 492 nm and emission filter of 620 nm. Raw run data were analysed using the TSA‐Craft tool.^[48]^

**H‐NMR spectroscopy**: To identify the oxidation site of N‐acetyl glucosamine oxidase by NMR analysis (Bruker Avance NEO 600, 600 MHz), a conversion with N‐acetyl Glucosamine was carried out. The reaction mixture contained 5 mM of N‐acetyl Glucosamine, 1 μM of enzyme in 50 mM of potassium phosphate buffer, pH 7.5. The reaction was performed at room temperature for one hour and quenched by heating at 95 °C for 10 minutes. The sample was then centrifuged for 10 minutes at 13500 rpm and the resulting supernatant was lyophilized overnight and resuspended in deuterium oxide 99 %. The standard reaction was performed under the same conditions without the enzyme.

**Growth of** ***E. coli*** **expressing HexNAcO**: Plasmids for expression of a HexNAcO (A3RXB7), and a control protein, peroxide producing lactate oxidase (LacO, A9QH69), were transformed into *E. coli* BL21(DE3) cells. Overnight cultures were set up in LB medium with 100 μg/mL carbenicillin at 30 °C. Next morning the OD_600_ of the cultures was set equal to 0.2, and 400 μL were used to inoculate 20 mL of LB medium with 100 μg/mL carbenicillin. For each plasmid, the inoculated medium was divided equally into two 50 mL and incubated at 37 °C with 200 rpm shaking. OD_600_ values of the cultures were recorded periodically (Thermo Scientific Genesys 10S spectrophotometer), and after 3.5 h from the start (OD_600_ around 0.8) one culture for each plasmid was induced with 0.5 mM IPTG, while one culture was left uninduced. OD_600_ values of the cultures were recorded periodically, up to 4 h post‐induction. After that time, cells were centrifuged, lysed as described above, and total lysate fractions were analysed by SDS‐PAGE.

### Computational methods

**Sequence selection**: Representative sequences were selected as described before.^[24]^ Briefly, sequences annotated to EC 1.1.3.X were downloaded from BRENDA DB (version 2017.1), and used for an all versus all BLAST analysis, followed by MCL clustering and multiple sequence alignment. For each cluster, sequences were iteratively selected using Shannon entropy so that each newly chosen sequence maximally increases the mutual information explained within each cluster. As a result, 185 sequences were selected, and the first 96 of them were tested in this study, to fit a 96‐well expression plate.

**Structure prediction models**: Homology model of HexNAcO was created using a RaptorX template‐based server, with a 2Y08A structure (tirandamycin oxidase TamL) as a template.^[49]^ Due to the lack of a homologous protein in PDB, the structural model of LCAO was created using AlphaFold Collab, a machine learning structure prediction approach.^[39]^

**Phylogenetic trees**: Protein BLAST search of non‐redundant protein sequences was performed, with a threshold of 40 % or more sequence identity covering a minimum 95 % of sequence. For these, species names of the host organisms were downloaded, omitting the strain names. Phylogenetic trees were created for these species using PhyloT, and visualised using iTOL.^[50]^

## Conflict of interest

The authors declare no conflict of interest.

## Supporting information

As a service to our authors and readers, this journal provides supporting information supplied by the authors. Such materials are peer reviewed and may be re‐organized for online delivery, but are not copy‐edited or typeset. Technical support issues arising from supporting information (other than missing files) should be addressed to the authors.

Supporting Information
