# Supplementary material for: Discovery of Two Novel Oxidases Using a High‐Throughput Activity Screen
Source: Chembiochem. 2021 Nov 18;23(2):e202100510. doi: 10.1002/cbic.202100510 (PMC9299179; doi:10.1002/cbic.202100510)
Supplement: Supplementary file 1 — Supporting Information [file CBIC-23-0-s001.pdf]

ChemBioChem

Supporting Information

Discovery of Two Novel Oxidases Using a High-Throughput Activity Screen

Elzbieta Rembeza, Alessandro Boverio, Marco W. Fraaije,* and Martin K. M. Engqvist*

Table S1. Proteins assayed in the “all-vs-all” experiment.

UniProt ID	Predicted EC group	Purified	Activity with substrate	Confirmed EC
A0A009NBJ7	1.1.3.6	FALSE	NA	NA
A0A011R8E6	1.1.3.15	FALSE	NA	NA
A0A073CBY9	1.1.3.15	TRUE	none	NA
A0A075HNX4	1.1.3.8	TRUE	dodecanol	1.1.3.20
A0A085FLP7	1.1.3.15	TRUE	none	NA
A0A087D1R1	1.1.3.15	TRUE	none	NA
A0A090SES2	1.1.3.15	FALSE	NA	NA
A0A0A1ZY49	1.1.3.15	FALSE	NA	NA
A0A0A8UQ43	1.1.3.15	TRUE	none	NA
A0A0B2PFM1	1.1.3.8	FALSE	NA	NA
A0A0D6UVV4	1.1.3.6	FALSE	NA	NA
A0A0D8HWN7	1.1.3.6	FALSE	NA	NA
A0A0D8HYB4	1.1.3.6	FALSE	NA	NA
A0A0E9C319	1.1.3.37	TRUE	none	NA
A0A0F0C8R9	1.1.3.15	TRUE	none	NA
A0A0F0KK17	1.1.3.6	FALSE	NA	NA
A0A0F4YNJ0	1.1.3.15	TRUE	none	NA
A0A0F8B231	1.1.3.20	FALSE	NA	NA
A0A0F8B4A0	1.1.3.15	FALSE	NA	NA
A0A0H6BK84	1.1.3.15	FALSE	NA	NA
A0A0J6V898	1.1.3.15	TRUE	none	NA
A0A0K2B175	1.1.3.41	TRUE	dodecanol, mannitol, xylitol	1.1.3.41
A0A0K3BPM4	1.1.3.15	TRUE	none	NA
A0A0L6Z980	1.1.3.15	TRUE	none	NA
A0A0M2H872	1.1.3.15	FALSE	NA	NA
A0A0M7LBC1	1.1.3.15	TRUE	none	NA
A0A0M7QHG7	1.1.3.21	FALSE	NA	NA
A0A0N1ND03	1.1.3.15	FALSE	NA	NA
A0A0P1DE25	1.1.3.15	TRUE	none	NA
A0A0P7WDX3	1.1.3.15	TRUE	none	NA
A0A0T9WLB5	1.1.3.6	TRUE	none	NA
A0A0U0XLD7	1.1.3.41	TRUE	none	NA
A0A0U0YMT3	1.1.3.6	FALSE	NA	NA
A0A0U1A0U5	1.1.3.41	TRUE	none	NA
A0A0U1AUR2	1.1.3.41	TRUE	none	NA
A0A0U1BXY3	1.1.3.6	FALSE	NA	NA
A0A0U5JSS4	1.1.3.15	TRUE	none	NA
A0A0W0SGJ0	1.1.3.15	FALSE	NA	NA
A2CCV2	1.1.3.15	FALSE	NA	NA
A2QTA3	1.1.3.38	TRUE	none	NA
A2Z8H1	1.1.3.20	FALSE	NA	NA
A3RXB7	1.1.3.45	TRUE	N-acetyl-D-glucosamine	1.1.3.29
A4HXL3	1.1.3.8	FALSE	NA	NA
A4YVE0	1.1.3.15	TRUE	none	NA
A9D099	1.1.3.15	FALSE	NA	NA
A9QH69	1.1.3.15	TRUE	glycolate	1.1.3.15
B1HZY7	1.1.3.15	TRUE	none	NA
B2IUG1	1.1.3.9	TRUE	none	NA
B3R195	1.1.3.9	TRUE	none	NA
B7N6P4	1.1.3.15	TRUE	none	NA
B9R845	1.1.3.9	FALSE	NA	NA
B9S0Y9	1.1.3.15	FALSE	NA	NA
B9ST70	1.1.3.15	FALSE	NA	NA
B9WMY6	1.1.3.37	TRUE	D-arabinono-1,4-lactone	1.1.3.37
C0XIJ3	1.1.3.15	TRUE	none	NA
C4WH31	1.1.3.37	TRUE	none	NA
C9Y9E7	1.1.3.15	FALSE	NA	NA
D3NRV6	1.1.3.15	FALSE	NA	NA
D4MUV9	1.1.3.15	TRUE	none	NA
D4N087	1.1.3.15	FALSE	NA	NA
D4XIR1	1.1.3.15	TRUE	none	NA
E0MUP1	1.1.3.8	TRUE	none	NA
E1UR06	1.1.3.8	FALSE	NA	NA
E3J7L2	1.1.3.6	TRUE	none	NA
F4CRU5	1.1.3.15	FALSE	NA	NA
F4G5A4	1.1.3.15	TRUE	none	NA
F8B3Q8	1.1.3.9	FALSE	NA	NA
F9EP79	1.1.3.21	TRUE	sn-glycerol-3-phosphate	1.1.3.21
F9ZK57	1.1.3.9	FALSE	NA	NA
G6F8W6	1.1.3.15	TRUE	none	NA
H0EAY8	1.1.3.6	FALSE	NA	NA

H0SIC1	1.1.3.15	TRUE	none	NA
K0NHF1	1.1.3.15	TRUE	none	NA
K4LGZ0	1.1.3.15	TRUE	none	NA
K4R330	1.1.3.6	FALSE	NA	NA
K9U6P8	1.1.3.6	TRUE	none	NA
L1KIC4	1.1.3.6	TRUE	cholesterol	1.1.3.6
M2Y0E7	1.1.3.15	FALSE	NA	NA
M4KHX7	1.1.3.15	TRUE	none	NA
P56216	1.1.3.38	TRUE	4-Hydroxybenzyl alcohol, vanillyl alcohol	1.1.3.38
P93762	1.1.3.5	TRUE	glucose, galactose	1.1.3.5
Q2LWG4	1.1.3.15	TRUE	none	NA
Q2YIX9	1.1.3.8	TRUE	none	NA
Q5WIP4	1.1.3.15	TRUE	none	NA
Q6MTY6	1.1.3.21	TRUE	sn-glycerol 3-phosphate	1.1.3.21
Q7WZ62	1.1.3.5	TRUE	none	NA
Q8EEB0	1.1.3.48	TRUE	none	NA
Q8XW09	1.1.3.15	FALSE	NA	NA
R7ZSU7	1.1.3.6	TRUE	none	NA
S0FWI7	1.1.3.15	FALSE	NA	NA
S5UC18	1.1.3.15	TRUE	none	NA
S7SZ03	1.1.3.15	FALSE	NA	NA
U4PUB6	1.1.3.15	TRUE	none	NA
V9Q617	1.1.3.6	FALSE	NA	NA
W9N2G4	1.1.3.20	TRUE	none	NA
X7YWP4	1.1.3.15	FALSE	NA	NA

Table S2. Substrates used in the “all-vs-all” experiment and the enzyme class (EC) they represent.

**Substrate**	**EC**
D-glucose	1.1.3.4, 1.1.3.5
D-galactose	1.1.3.5, 1.1.3.9
cholesterol	1.1.3.6
3-methoxybenzyl alcohol	1.1.3.7
L-gulono-1,4-lactone	1.1.3.8
L-sorbose	1.1.3.10
pyridoxine	1.1.3.12
ethanol	1.1.3.13
1,2-Dihydroxybenzene	1.1.3.14
glycolic acid	1.1.3.15
choline	1.1.3.17
2-hexanol	1.1.3.18
dodecyl alcohol	1.1.3.20
sn-glycerol 3-phosphate	1.1.3.21
thiamine	1.1.3.23
adenosine	1.1.3.28
N-acetyl-D-glucosamine	1.1.3.29
polyvinyl alcohol	1.1.3.30
D-arabinono-1,4-lactone	1.1.3.37
4-Hydroxybenzyl alcohol	1.1.3.38
vanillyl alcohol	1.1.3.38
mannitol	1.1.3.40
xylitol	1.1.3.41

Table S3. Kinetic values obtained for the N-acetyl-D-hexosamine oxidase A3RXB7 (+/- standard error of three replicates).

**substrate**	**Km (mM)**	**kcat (s^{-1})**	**kcat/Km (M^*s^{-1})**
GlcNAc	0.26 ± 0.03	5.67 ± 0.16	21807.7
GalNAc	0.32 ± 0.02	4.46 ± 0.08	13937.5
ManNAc	181.76 ± 36.16	2.23 ± 0.28	12.3
diacetylchitobiose	18.70 ± 1.84	0.84 ± 0.04	44.9
D-glucosamine	4.54 ± 0.57	0.35 ± 0.01	77.1
D-galactosamine	1.35 ± 0.22	0.62 ± 0.02	459.3
D-mannosamine	65.70 ± 10.21	0.011 ± 0.001	0.2
D-glucose	216.62 ± 34.32	0.16 ± 0.01	0.7
D-galactose	101.74 ± 12.87	0.19 ± 0.01	1.9
D-mannose	118.48 ± 11.16	0.14 ± 0.01	1.2

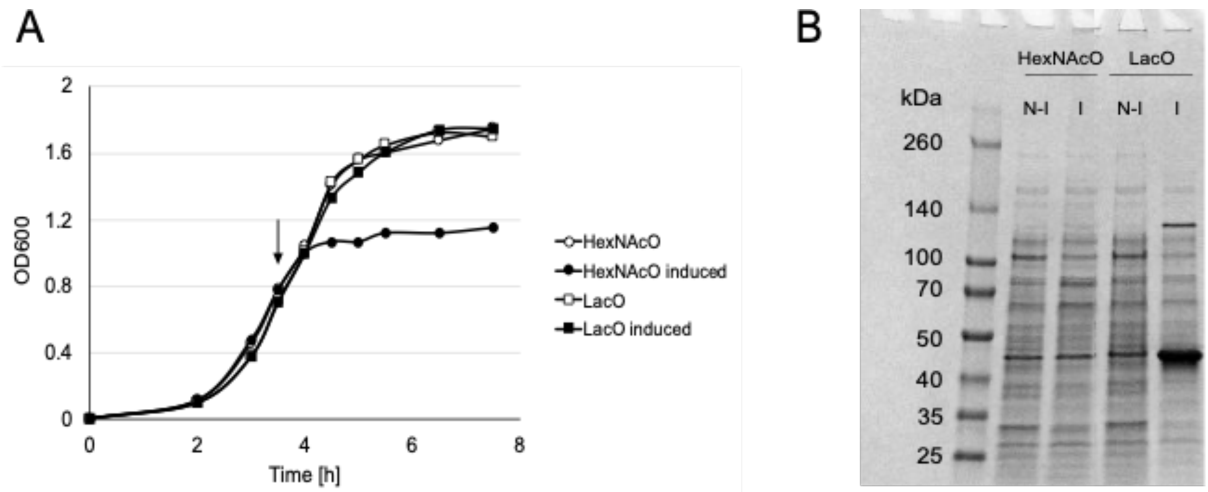

Fig. S1 Expression of HexNAcO in *E. coli* cells. HexNAcO - A3RXB7 from *R. solanacearum*, LacO - A9QH69 from *S. iniae.* (A) Growth profile of *E. coli* cells carrying oxidase expression plasmids, induction time with 0.5mM IPTG indicated with an arrow. (B) SDS-PAGE gel with total lysates of *E. coli* cultures. “N-I” - non-induced cultures, “I” - cultures induced with 0.5mM IPTG.

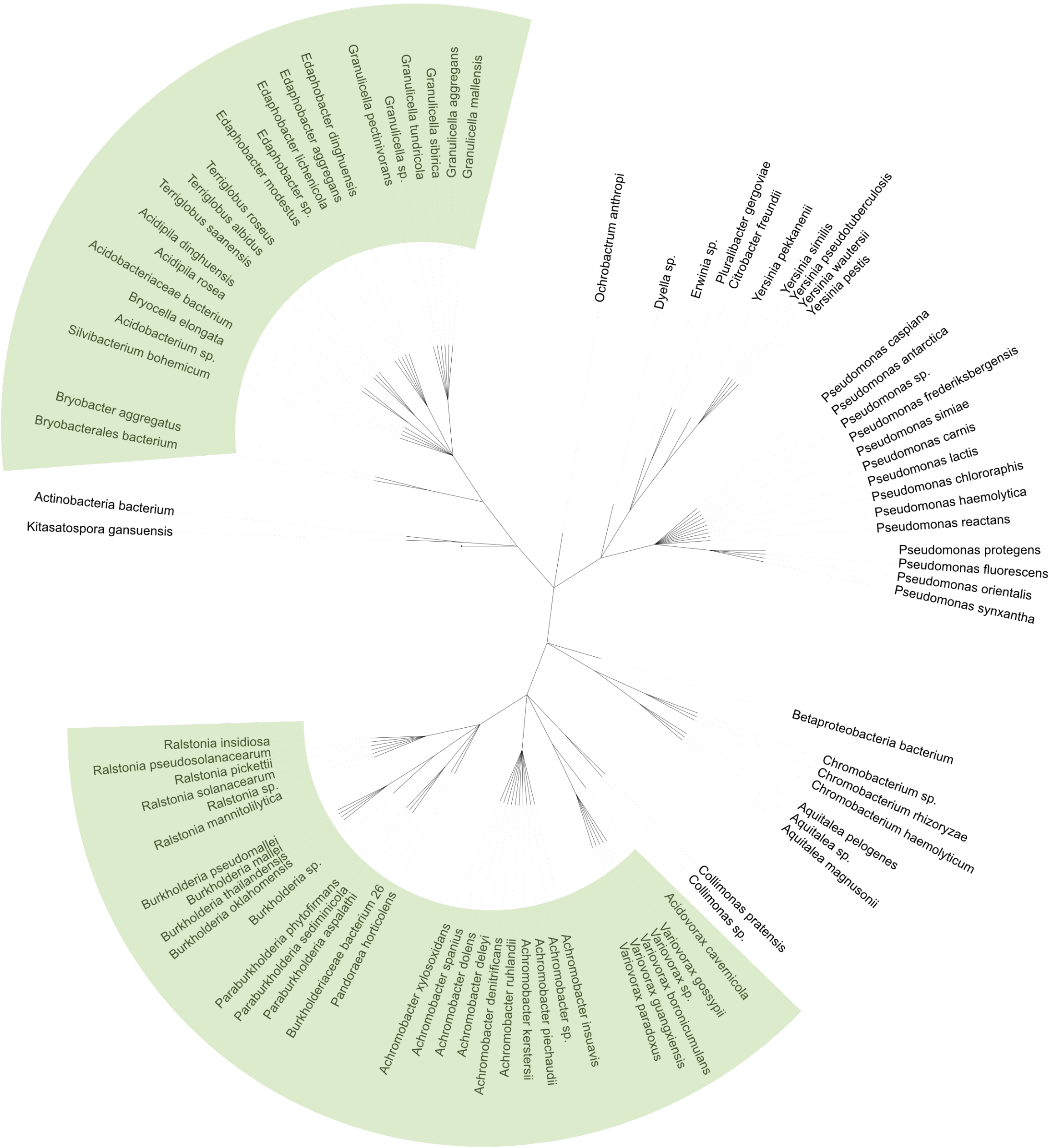

Fig. S2 | Phylogenetic tree of bacterial species containing a homologue of HexNAcO from *R. solanacearum.* Highlighted in green are the species containing homologues with down to 50% sequence identity, in white are the species containing homologues with sequence identity 40-49%.

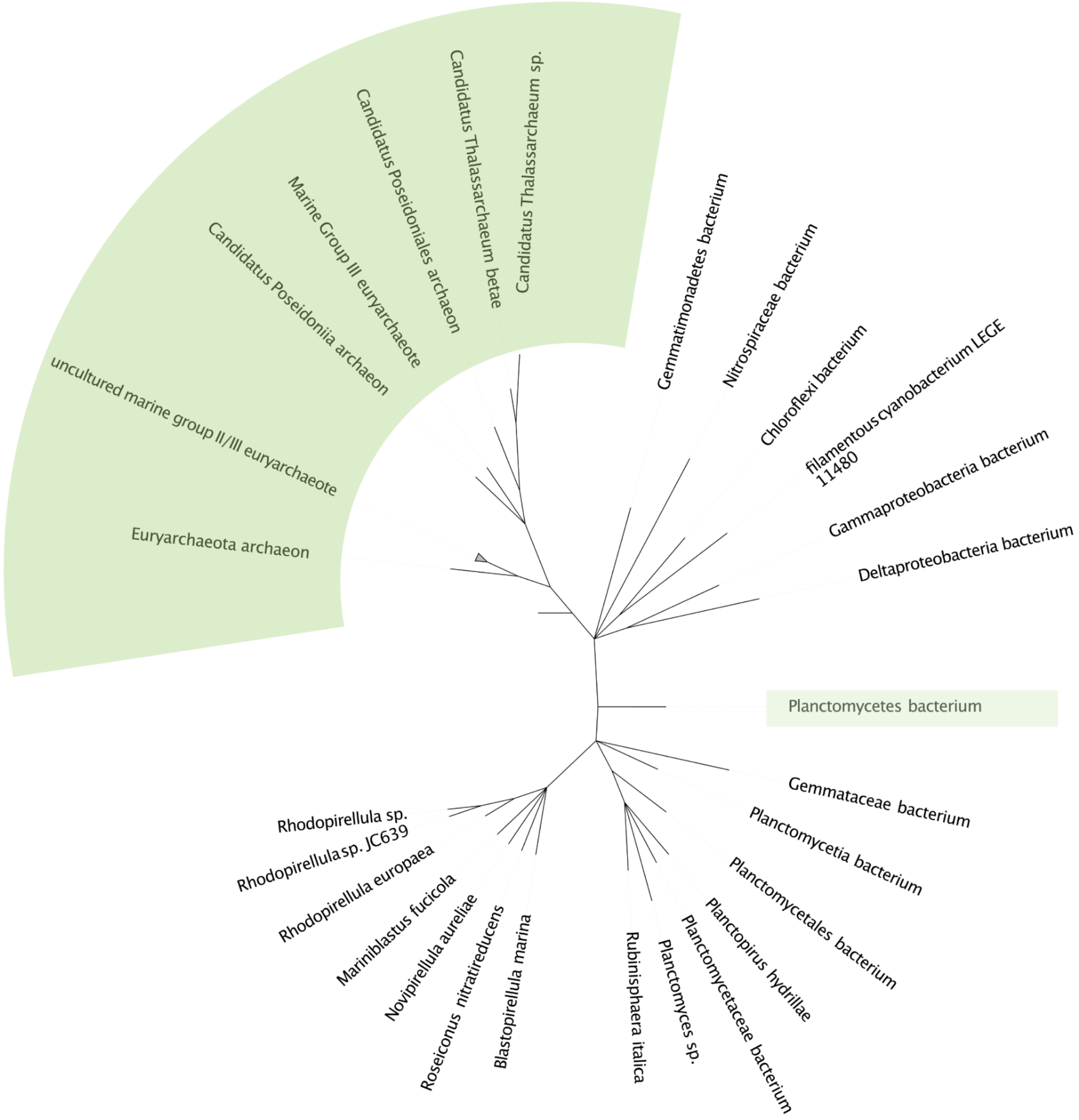

Fig. S3 | Phylogenetic tree of species containing a homologue of archaeal LCAO. Highlighted in green are the species containing homologues with down to 50% sequence identity, in white are the species containing homologues with sequence identity 40-49%.
